# Assessment of the environmental risk factors for a gastric ulcer in northern Ghana

**DOI:** 10.11604/pamj.2016.25.160.8531

**Published:** 2016-11-15

**Authors:** Stephen Tabiri, Prosper Akanbong, Braimah Baba Abubakari

**Affiliations:** 1 University for Development Studies, School of Medicine and Health Sciences, Tamale Teaching Hospital, Department of Surgery, Tamale; 2 University for Development Studies, School of Medicine and Health Sciences, Tamale Teaching Hospital, Department of Internal Medicine, Tamale, Ghana

**Keywords:** Gastric ulcer disease, dyspepsia, H. pylori infection, endoscopy

## Abstract

Numerous risk factors have been implicated in the development of a gastric ulcer. Common risk factors are *Helicobacter pylori* infection, chronic non-steroidal anti-inflammatory intake, and alcohol consumption. The aim of the current study was to identify environmental risk factors for a gastric ulcer in northern Ghana. The data for this retrospective study were obtained from 2035 patient records from the Minimal Access Therapy and Operative Endoscopy unit of the Tamale Teaching Hospital in Tamale, Ghana from 2010 to 2014. A separate questionnaire was administered to assess the environmental risk factors. The rapid urease test was used to determine the presence of *H. pylori.* The Statistical Package for Social Sciences version 20.0 was used to analyse the data. Univariate and bivariate analyses were performed, and the results were presented in tables provided. The Chi-square values of the bivariate analysis were considered statistically significant when P < 0.05. Bivariate analysis revealed a strong association between gastric ulcer and various risk factors such as smoking (P = 0.001, χ^2^ = 27.3), fasting (P = 0.001, χ^2^ = 42.6), H. pylori infection (P = 0.01, χ^2^ = 19.9), and alcohol consumption (P = 0.001, χ^2^ = 30.6). There was no association between the traditional herbal preparation usage (P = 0.251, χ^2^ = 1.8) and the gastric ulcer. Environmental risk factors responsible for the development of a gastric ulcer in people of the northern part of Ghana show a similar pattern to other geographical regions of the world.

## Introduction

Dyspepsia is a nonspecific term that denotes upper abdominal discomfort that is thought to arise from the upper gastrointestinal (GI) tract. A gastric ulcer is a common cause of dyspepsia that encompasses a variety of more specific symptoms including epigastric discomfort or pain, bloating, anorexia, early satiety, belching or regurgitation, nausea, and heartburn. The gastric ulcer has been found in 4.3% of patients with dyspeptic symptoms in the central part of Ghana [1]. Numerous risk factors have been implicated in the development of a gastric ulcer. Common environmental risk factors attributed to the development of gastric ulcer include *Helicobacter pylori* infection, chronic non-steroidal anti-inflammatory (NSAID) intake, and alcohol consumption. Environmental factors play a vital role in the development of a gastric ulcer. The aim of this study was to identify environmental risk factors responsible for the development of a gastric ulcer in patients in northern Ghana undergoing endoscopic diagnosis.

## Methods

**Figure 1:**
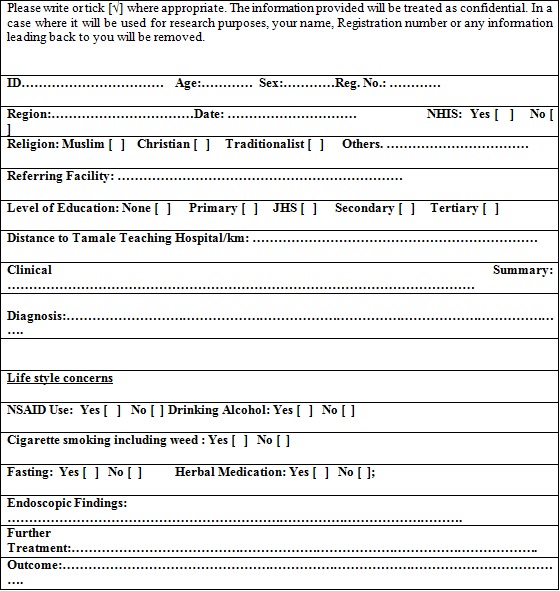
Questionnaires to identify risk factors of gastric ulcer

The data for this retrospective study were obtained from patient records from the Minimal Access Therapy and Operative Endoscopy unit of the Tamale Teaching Hospital in Tamale, Ghana from 2010 to 2014. All of the patients who received an upper GI endoscopic diagnosis at the unit were included in the study. Patients were either referred to the hospital from other facilities or reported directly to the hospital. Those patients who underwent colonoscopy were excluded. The objectives of the study were explained to the patients on the appointed day, and a written consent form was obtained from each patient. The study was approved by the Tamale Teaching Hospital Ethical Review Committee (TTHERC/18/11/15/07. A separate questionnaire was administered to patients by an endoscopy nurse before the procedure to assess risk factors for a gastric ulcer (Figure 1). Information on the socio-demographic status, GI symptoms and clinical diagnosis were obtained from patients' folders. Gastric biopsies for rapid urease test were performed during endoscopy for detection of H. pylori. The endoscopic findings and the results of the rapid urease test were recorded. Data collected from patients' records included socio-demographic status, symptoms presented, and the results of clinical and endoscopy diagnoses. Statistical Package for Social Sciences (SPSS) version 20.0 was used to analyse the data. Univariate and bivariate analyses were performed to ascertain environmental risk factors for gastric ulcer. The Chi-square values of the bivariate analysis were considered statistically significant when P < 0.05.

## Results

Table 1: A total of 2035 patients were included in the study. Some patients had more than one endoscopy finding; therefore, the total number of endoscopy findings was 3176. The age range was 2-89 years, and a mean age was 40.2 ± 16.7 years (mean ± SD). A total of 1037 men (51.0%) and 998 women (49.0%) were included in the study. The largest number of patients was in the age group 31-40 years (680/2035, 34.4%. The least number of patients was in the age group 2-15 years (35/2035, 1.7%. The epigastric pain was the most frequent major symptom (1551/2035, 76.2%) followed by abdominal pain (1002/2035, 49.2%). Other significant symptoms such as heartburn, effortless vomiting, hematemesis, and dysphagia, were present in 13.4% (273/2035), 10.6% (215/2035), 7.5% (153/2035), and 6.5% (132/2035) patients, respectively. The peptic ulcer disease (PUD) clinical diagnosis was most common (1451/2035, 71.3%), followed by gastro-reflux disease (GORD) (184/2035, 9.0%), upper GI bleeding (144/2035, 7.1%), gastric cancer (136/2035, 6.7%), and oesophageal cancer (61/2035, 3.0%). A total of 1259/2053 (61.9%) patients tested positive for *H. pylori* infection.

**Table 1:** Socio-demographic status, main symptoms, clinical diagnosis, endoscopy findings and *Helicobacter pylori* infection in patients with gastric ulcer

Variable		Frequency	Percentage
**Sex** **(*n* = 2035)**	Male	998	49.0
	Female	1037	51.0
**Age in years** **(*n* = 2035)**	0-20	35	1.7
	21-30	652	32.0
	31-40	680	34.4
	41-50	399	19.6
	51-60	199	9.8
	>60	70	3.4
**Major symptoms** **(*n* = 3724)**	Epigastric pain	1551	41.6
	Abdominal pain	1002	26.9
	Heartburn	273	7.3
	Persistent vomiting	215	5.8
	Hematemesis	153	4.1
	Dysphagia	132	3.5
	Others	398	10.7
**Clinical diagnosis** **(*n* = 2092)**	PUD	1505	71.9
	GORD	184	9.0
	Upper GI bleeding	144	6.9
	Gastric cancer	136	6.5
	Oesophageal cancer	61	2.9
	Others	62	3.0
**Endoscopy diagnosis** **(*n* = 3176)**	Gastritis	892	28.1
	Gastric ulcer	615	19.4
	GORD	586	18.4
	Gastric cancer	263	8.3
	Duodenal ulcer	226	7.1
	Oesophageal cancer	191	6.0
	Upper GI bleeding	104	3.3
	Normal	112	3.5
	Others	187	5.9
*H. pylori* infection (*n* = 2035)		1286	63.2

PUD- Peptic ulcer disease; GORD - Gastro-oesophageal reflux disease; GI - Gastrointestinal

Of 2035 patients, 1344 (66.0%) marked NSAID on the questionnaire; 1812 (89.0%) patients admitted alcohol intake greater than 5 units/day. Herbal usage for medicinal purposes (traditional concoction) was used by 822 (40.4%) patients. Cigarettes and cannabis were used by 183 (8.9%) patients. Fasting or irregular eating was present in 1126 (55.3%) cases.

Table 2: Bivariate analysis revealed a strong association between gastric ulcer and the following risk factors: smoking of cigarette including weeds (P = 0.001, χ^2^ = 27.3), fasting (P = 0.001, χ^2^ = 42.6), *H. pylori* infection (P = 0.01, χ^2^ = 19.9), and alcohol consumption (P = 0.001, χ^2^ = 30.6). There was no association between herbal usage and gastric ulcer (P = 0.251, χ^2^ = 1.8).

**Table 2:** environmental risks factors for development of gastric ulcer in patients in northern Ghana

Variable (*n* = 2035)	Frequency	Percentage	χ^2^	*P*
Alcohol consumption >5 units/day			30.6	0.001*
Yes	1812	89.0		
No	223	11.0		
Smoking:			27.3	0.001*
Yes	183	9.0		
No	1852	91.0		
Long fasting (irregular eating habit):			42.6	0.001*
Yes	1126	55.3		
No	909	44.7		
NSAID:			24.9	0.001*
Yes	691	34.0		
No	1344	66.0		
CLO test result:			39.3	0.01
Positive	1286	63.2		
Negative	749	36.8		
Traditional herbal preparation:			1.8	0.251
Yes	822	40.4		
No	1213	59.6		

NSAID - Non-steroidal anti-inflammatory intake; CLO - Campylobacter-like organism

* *P <* 0.05 statistically significant

## Discussion

Dyspepsia is a common affliction with an estimated prevalence of up to 40% in the general population worldwide [2]. It accounts for 25-47% of patients refer for upper GI endoscopy in East Africa [3–5]. The patients included in the study were between 2 and 89 years old; the majority were 31-45 years old. These findings differ from Veldhuyzen et al. [6] who found dyspeptic symptoms predominantly in people <20 years. In our study, the dyspeptic symptoms were predominantly found in older patients. This could be attributed to lifestyle choices such as irregular eating habits, cigarette smoking, and chronic NSAID intake prevailing in the older group in Ghana. Ohene-Yeboah et al [7] found that NSAID, herbal medicines or concoctions are commonly abused by patients with gastric perforation [6, 7]. In the current study, the incidence of epigastric pain was 41.6% which is consistent with the previous report [8]. Clinical diagnosis of PUD was 71.9% in our series, similar to earlier findings [9].

Common causes of dyspepsia symptoms include gastric ulcer, GORD, functional disorders (non-ulcer dyspepsia), malignancy, and age <45 years [10–14]. According to Numans et al. [15], *H. pylori* infection plays a crucial role in the pathogenesis of gastric ulcer. In patients suffering from gastric ulcers, 70% - 80% are H. pylori positive on rapid urease test of gastric biopsy at endoscopy [16, 17]. PUD has been associated with smoking, *H. pylori* infection, and chronic NSAID intake [18–20]. Numerous factors are implicated in the pathophysiology of gastric ulcer. These include smoking habits, alcohol consumption, coffee drinking, and familial occurrences of peptic ulcers in patients with a gastric or duodenal ulcer. Epidemiologic studies suggest that smokers are about twice as likely to develop gastric ulcers compare to non-smokers [21]. Bivariate analyses identified smoking, *H. pylori* infection, fasting, alcohol consumption, and chronic intake of NSAID as risk factors for gastric ulcer which is similar to the findings in other parts of the world.

## Conclusion

Previously identified environmental risk factors for gastric ulcer development show a similar pattern in patients of the northern part of Ghana compared with other geographical regions of the world.

### What is known about this topic

- Risk factors of gastric ulcer in other population settings;
- Causes of peptic ulcer disease in different population settings in Ghana.

### What this study adds

- Identification risk factors of gastric ulcer in Northern Ghana for the first time;
- Describes association of the risk with endoscopy findings in this population;
- Knowledge to the problem of gastric ulcer.
